# Systematic review: the diagnosis and staging of non-alcoholic fatty liver disease and non-alcoholic steatohepatitis

**DOI:** 10.1111/j.1365-2036.2010.04556.x

**Published:** 2010-12-29

**Authors:** J K Dowman, J W Tomlinson, P N Newsome

**Affiliations:** * Centre for Liver Research, 5th floor, Institute of Biomedical Research, University of BirminghamEdgbaston, Birmingham B15 2TT, UK; † The Liver Unit, Queen Elizabeth Hospital BirminghamEdgbaston, Birmingham B15 2TH, UK; ‡ Centre for Endocrinology, Diabetes and Metabolism, University of BirminghamEdgbaston, Birmingham B15 2TT, UK

## Abstract

**Background:**

Non-alcoholic fatty liver disease (NAFLD) has become the most prevalent cause of liver disease in Western countries. The development of non-alcoholic steatohepatitis (NASH) and fibrosis identifies an at-risk group with increased risk of cardiovascular and liver-related deaths. The identification and management of this at-risk group remains a clinical challenge.

**Aim:**

To perform a systematic review of the established and emerging strategies for the diagnosis and staging of NAFLD.

**Methods:**

Relevant research and review articles were identified by searching PubMed, MEDLINE and EMBASE.

**Results:**

There has been a substantial development of non-invasive risk scores, biomarker panels and radiological modalities to identify at-risk patients with NAFLD without recourse to liver biopsy on a routine basis. These modalities and algorithms have improved significantly in their diagnosis and staging of fibrosis and NASH in patients with NAFLD, and will likely impact on the number of patients undergoing liver biopsy.

**Conclusions:**

Staging for NAFLD can now be performed by a combination of radiological and laboratory techniques, greatly reducing the requirement for invasive liver biopsy.

## Introduction

Non-alcoholic fatty liver disease (NAFLD) encompasses a spectrum of disease ranging from simple steatosis, to inflammatory steatohepatitis (NASH) with increasing levels of fibrosis and ultimately cirrhosis. NAFLD is closely associated with obesity and insulin resistance, and is now recognised to represent the hepatic manifestation of the metabolic syndrome. Since the term NASH was first coined by Ludwig *et al.* in 1980,^1^ the prevalence of NAFLD has risen rapidly in parallel with the dramatic rise in population levels of obesity and diabetes,^2^ resulting in NAFLD now representing the most common cause of liver disease in the Western world.^3^

Despite recent advances in elucidating the complex metabolic and inflammatory pathways involved in NAFLD, the pathogenesis of steatosis and progression to steatohepatitis and fibrosis/cirrhosis is not yet fully understood.^4^, ^5^ While steatosis alone appears to be associated with a relatively benign prognosis,^6^ factors known to be involved in progression to more advanced and clinically relevant disease include inflammatory cytokines/adipokines, mitochondrial dysfunction and oxidative stress.^7^ Insulin resistance causes impaired suppression of adipose tissue lipolysis, leading to increased efflux of free fatty acids (FFA) from adipose tissue to the liver.^8^ Hyperinsulinaemia also promotes hepatic *de novo* lipogenesis, which is markedly increased in NAFLD patients compared with normal individuals.^9^ It is now recognised that FFA promote insulin resistance, inflammation and oxidative stress,^10^, ^11^ and thus rather than being harmful, hepatic triglyceride accumulation may actually be protective by preventing the harmful effects of FFA.^12^ The important role of oxidative stress mechanisms, pro-inflammatory cytokines such as TNFalpha and interleukin 6, and adipokines such as leptin (proinflammatory and pro-fibrotic), and adiponectin (anti-inflammatory and insulin-sensitising), in promoting NASH are also becoming increasingly delineated.^5^ However, evidence that only a minority of patients with NAFLD progress to more advanced stages of NASH suggests that disease progression is likely to depend on a complex interplay between such factors and underlying genetic predisposition.^4^, ^7^

The causes, epidemiology and natural history of NAFLD will be covered briefly, before discussing the established and emerging means of assessing and staging patients with NAFLD.

### Causes of NAFLD

In the great majority of cases, NAFLD arises in association with one or more features of the metabolic syndrome, namely insulin resistance, glucose intolerance or diabetes, central obesity, dyslipidaemia and hypertension.^13^–^15^ However, after exclusion of a history of significant alcohol intake, which is conventionally <20 g/day,^16^ other causes of steatosis which should be considered include nutritional causes, e.g. rapid weight loss and total parenteral nutrition, rare metabolic disorders and drug-induced steatosis. Commonly implicated agents include glucocorticoids, amiodarone, synthetic oestrogens and highly active antiretroviral drugs (HAART).^16^–^18^ Steatosis is also frequently associated with hepatitis C, particularly genotype 3, and endocrine disorders such as polycystic ovary syndrome (PCOS),^19^, ^20^ hypopituitarism^21^ and hypothyroidism.^22^

### Epidemiology

The prevalence of NAFLD is estimated to be between 20% and 30% in Western adults,^23^, ^24^ rising to 90% in the morbidly obese.^25^ NASH, the more advanced and clinically important form of NAFLD, is less common, with an estimated prevalence of 2–3% in the general population^16^ and 37% in the morbidly obese.^25^ Of concern, NAFLD now affects 3% of the general paediatric population, rising to 53% in obese children,^26^, ^27^ with considerable implications for future disease burden. Steatosis was present in 70% of a large unselected cohort of patients with type 2 diabetes.^28^

Non-alcoholic fatty liver disease affects all ethnic groups, although prevalence appears to be higher in Hispanic and European Americans compared with African-Americans. This difference remains after controlling for insulin resistance and obesity^23^, ^29^ and may be related to ethnic differences in lipid metabolism.^23^, ^30^

### Natural history

Patients with a diagnosis of NAFLD have been shown across several studies to have a worse outcome when compared with an age and sex-matched general population.^31^ Of note, the excess mortality in this group is attributable to both cardiovascular and liver-related causes.^32^, ^33^ Since the description in 1999 of the prognostic relevance of different histological types of NAFLD,^34^ several subsequent studies have demonstrated that the presence of just simple steatosis, with no inflammation or fibrosis, is associated with a similar overall and liver-related mortality to that of an age and gender matched general population. This reinforces the need to stratify patients with NAFLD into simple steatosis or more advanced disease. More advanced disease can be defined as advancing levels of fibrosis and/or the presence/level of inflammation and hepatocyte ballooning. This distinction is pertinent as cohort studies thus far have only identified advanced fibrosis, and not inflammation, as a predictor of worse clinical outcome.^32^ This may be a type 2 error reflecting small sample sizes, or it may be attributed to additional factors such as PNPLA3 polymorphisms^35^ regulating the development of fibrosis.

## Methods

A systematic literature search was performed to identify studies assessing methods for the diagnosis and staging of NAFLD/NASH. Relevant articles were identified by searching the PubMed database, MEDLINE and EMBASE, limited to articles published in the English language but not date-restricted. Search terms included fatty liver, NAFLD, NASH, steatosis, AND biomarkers, non-invasive, diagnosis, assessment, staging. Additional searches were also made for each of the individual methods described, e.g. NAFLD fibrosis score, transient elastography, Fibroscan, Fibrotest etc. Selected articles referenced in these publications were also examined.

## Inclusion criteria

Studies were included if:

- they were meta-analyses, systematic reviews or primary studies of one or more relevant diagnostic/staging tool;
- they included at least 30 subjects, to reduce the risk of including underpowered studies;
- liver biopsy was used as the reference standard;
- the diagnosis of NAFLD had been established with exclusion of other causes of liver disease.

### Exclusion criteria

Studies were excluded if:

- publications were not in English;
- data on disease stage e.g. fibrosis stage, was not identifiable;
- they were only presented in abstract form.

Using the search strategy described above, approximately 150 articles were considered. Following review, 68 articles met the selection criteria and were included in the analyses.

### Data extraction

JD performed the data extraction, which was then checked by the remaining authors (PN and JT).

## NAFLD: making the diagnosis

**Figure 1:**
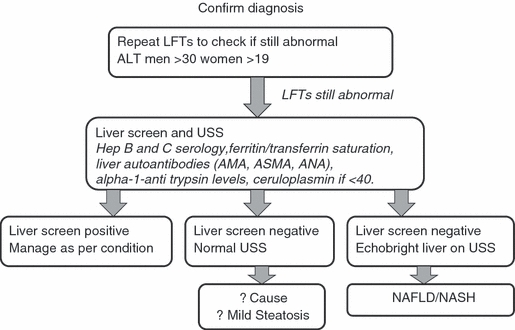
Making the diagnosis of NAFLD.

The diagnosis of NAFLD should be strongly suspected in the presence of features such as obesity, diabetes and obstructive sleep apnoea (OSA); however, other causes should always be considered before attributing abnormal liver function tests (LFTs) to NAFLD alone (Figure 1). Alternative diagnoses which should be excluded by history and serological testing include the viral hepatitides, excess alcohol consumption, haemochromatosis, autoimmune liver disease, alpha-1 antitrypsin deficiency, Wilson's disease and drug-induced liver dysfunction.

The majority of patients with NAFLD are asymptomatic and the diagnosis suspected after finding elevated transaminases on routine testing. Hepatic steatosis is also a frequent incidental finding on ultrasound scan (US) performed for other reasons such as suspected gallstone disease. The most common symptoms are right upper quadrant discomfort and fatigue, although the latter may also be caused by OSA which is frequently observed in the typically obese population with NAFLD. Hepatomegaly is the most common clinical finding, with signs of chronic liver disease rarely present in the absence of cirrhosis. A recent study reported the novel finding that increased dorsocervical lipohypertrophy was the anthropometric parameter most strongly associated with severity of steatohepatitis.^36^

Although NAFLD is often diagnosed after the finding of mildly abnormal LFTs, more than two thirds of patients have normal aminotransferase levels at any given time^37^ and the entire histological spectrum of NAFLD can be observed in patients with normal alanine aminotransferase (ALT) values.^38^, ^39^ ALT is usually greater than aspartate aminotransferase (AST), and rarely more than three times the upper limit of normal. An AST:ALT ratio greater than 1.0 suggests the presence of more advanced disease.^40^ Alkaline phosphatase can be slightly elevated but is rarely the only liver function test abnormality.^41^ Gamma-glutamyltransferase (GGT) is frequently elevated and may also be a marker of increased mortality.^42^, ^43^ Low albumin and hyperbilirubinaemia indicate advanced liver disease and are not otherwise features of NAFLD.^44^ Iron studies may show an elevated ferritin in up to 50% of patients and elevated transferrin saturation in approximately 10%.^40^ However, such findings do not appear to correlate with elevated hepatic iron concentration, and the role of hepatic iron in the pathogenesis of NASH remains unclear.^45^

The Fatty Liver Index (FLI) was developed as a simple algorithm to predict fatty liver on USS in the general population.^46^ The FLI uses four variables of BMI, waist circumference, GGT and serum triglyceride levels, and achieved an accuracy of 0.84 in detecting fatty liver.^46^ The FLI has since been utilised by several groups in population studies of NAFLD.^47^–^49^ Ultrasound (USS) is a commonly used test in patients with suspected NAFLD, with steatosis typically appearing as a hyperechogenic liver. A recent study examined the accuracy of USS in 235 patients with suspected liver disease who underwent liver biopsy, and showed a sensitivity of 64% and specificity of 97%, rising to 91% and 93% respectively in patients with at least 30% steatosis.^50^ However, the presence of morbid obesity considerably reduces sensitivity and specificity.^51^ USS is unable to quantify the amount of fat present or provide any staging of disease,^52^ and is operator-dependent with significant intra- and inter-observer variability.^53^

## Staging of NAFLD

Having made a diagnosis of NAFLD, the next step is to determine the severity, as that provides important information on prognosis. Historically this has required liver biopsy, although there have been many recent advances which allow non-invasive management for many patients. When staging patients with NAFLD, there are two aspects to consider; (i) the level of fibrosis and (ii) the level of inflammation/ballooning (Table 1).

**Table 1:** Methods for assessing fibrosis and NASH

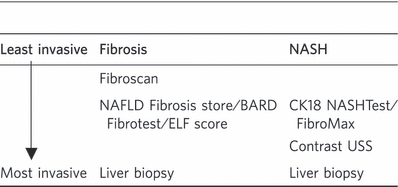

The histological spectrum of NAFLD ranges from simple steatosis through steatohepatitis to fibrosis and cirrhosis. There are no pathological changes which can definitively distinguish NAFLD from alcoholic liver disease (ALD), thus an accurate alcohol history is essential to distinguish between these two common conditions.^54^ The histological changes in NAFLD are mainly parenchymal and in a perivenular location, although portal and periportal lesions may occur.^54^ Simple steatosis is usually macrovesicular resulting from accumulation of triglycerides within hepatocytes.^44^ Features of steatohepatitis include hepatocellular injury, characterised by ballooned hepatocytes, with inflammation and fibrosis.^54^ Mitochondrial abnormalities may occur in NASH, but rarely in simple steatosis,^11^ supporting a role for mitochondrial defects in the pathogenesis of NAFLD-related liver injury.^54^, ^55^ The typical histological features of steatosis and inflammation often disappear in advanced disease,^56^, ^57^ thus many cases of ‘cryptogenic’ cirrhosis are likely caused by NASH.^56^–^58^ Hepatocellular carcinoma is a well-recognised complication of NASH-related cirrhosis,^59^, ^60^ but can also be associated with precirrhotic NAFLD.^61^, ^62^ Several systems have been proposed for the histological assessment of NAFLD, of which the Kleiner NAFLD activity score (NAS)^63^ is probably the most well established. The NAS provides a composite score based on the degree of steatosis (0–3), lobular inflammation (0–3) and hepatocyte ballooning (0–2), with an additional score for fibrosis. A score of ≥5 suggests probable or definite NASH, and <3 indicates that NASH is unlikely.^63^ However, although liver biopsy currently remains the gold standard for diagnosis of NASH, limitations of this technique include intra-observer variation^63^, ^64^ and sampling variability,^65^, ^66^ with features such as fibrosis often not uniformly distributed.^54^

### Non-invasive assessments of NAFLD severity

Such assessments can provide information on the amount of liver fibrosis and/or the presence of NASH, features which are usually, but not always, found together. The focus on fibrosis is based on cohort studies which demonstrate that fibrosis, rather than inflammation, predicts outcome. Several non-invasive diagnostic panels and scoring systems have been developed with varying diagnostic utility. The uneven distribution of fibrosis throughout the liver in NAFLD indicates that such scoring systems may potentially represent a more accurate reflection of global liver fibrosis severity than is permitted by the current gold standard liver biopsy,^67^ which samples only 1/50 000th of the organ and is prone to significant sampling error.^65^, ^66^

#### Assessment of fibrosis

*(i) Demographic factors and simple blood tests:* Several diagnostic panels have been developed to facilitate the non-invasive assessment of NAFLD and differentiation between different stages of disease. These are generally based on a number of laboratory measurements, often in combination with clinical parameters such as age, sex and BMI. Such scoring systems have generally demonstrated greater utility in the detection of advanced fibrosis than intermediate and early stages of fibrosis, a group potentially more likely to benefit from therapeutic interventions.^37^

The BARD score is a simple scoring system designed to identify NAFLD patients with a low risk of advanced disease. It combines three variables of BMI, AST/ALT ratio (AAR) and the presence of diabetes into a weighted sum (BMI ≥28 = 1 point, AAR of ≥0.8 = 2 points, DM = 1 point), to generate a score from 0 to 4. In the original study, a score of 2–4 was shown to be associated with an odds ratio for advanced fibrosis of 17 and a negative predictive value of 96%.^68^ A further study of the BARD score in 138 patients with biopsy-proven NAFLD revealed an area under the receiver operating curve (AUROC) of 0.67 (95% CI, 0.56–0.77), with sensitivity, specificity, positive predictive value (PPV) and negative predictive value (NPV) of 51%, 77%, 45% and 81% respectively.^69^ In a recent study including 145 patients with biopsy-proven NAFLD, McPherson *et al.* compared the diagnostic performance of five simple non-invasive tests [BARD score, NAFLD fibrosis score, FIB-4 score, AST to platelet ratio index (APRI) and ALT/AST ratio], for the identification of NASH-related advanced fibrosis. Here the BARD score demonstrated an AUROC of 0.77, with sensitivity 89%, specificity 44%, NPV 95% and PPV 25%.^70^ The BARD score was also validated in a Polish NAFLD cohort, where an NPV of 97% was demonstrated,^71^ but appeared less useful in a Japanese cohort, where the AUROC was 0.73 with NPV 77%.^72^ The BARD score is easily calculated and thus represents a simple tool for excluding the presence of advanced fibrosis in NAFLD patients.

The AST-to-platelet ratio index (APRI),^73^ AST/ALT ratio,^74^ and FIB-4 score^75^ have previously demonstrated utility in the non-invasive assessment of fibrosis in a number of chronic liver diseases. Several recent studies have also examined the role of these markers in NAFLD, as will be described.

The APRI was originally developed for use in chronic hepatitis C,^73^ but its utility in NAFLD has since been studied by a number of groups. Using this score, Cales *et al.* demonstrated an AUROC of 0.866 for significant fibrosis, 0.861 for severe fibrosis and 0.842 for cirrhosis in a study of 235 NAFLD subjects.^76^ However, significantly lower values were obtained in other studies, where AUROCs of 0.564 for significant fibrosis, 0.568 for advanced fibrosis,^77^ and 0.786 for predicting cirrhosis^78^ were demonstrated. In their study of 145 NAFLD patients, McPherson *et al.* reported an AUROC of 0.67 for the diagnosis of advanced fibrosis.^70^

The AST/ALT ratio (AAR) is calculated using two widely available laboratory liver function tests. In addition to its utility as an individual marker, the AAR is also a component of several other fibrosis scoring systems including the NAFLD Fibrosis score and BARD score. Despite its simplicity, using a cut-off of 0.8 McPherson *et al.* demonstrated an AUROC of 0.83, with sensitivity 74%, specificity 78% and NPV of 93% for the diagnosis of advanced fibrosis in NAFLD using the AAR.^70^ The United States Nonalcoholic Steatohepatitis Clinical Research Network (NASH CRN) recently investigated the utility of readily available clinical and laboratory variables to predict histological severity of NASH in >600 patients with biopsy-proven NAFLD. In this study, a combination of serum AST, ALT and the AAR performed only modestly (AUROC 0.59) for predicting steatosis, but was able to predict cirrhosis with an AUROC of 0.81. However, the addition of demographic data, comorbidities and several other routinely measured laboratory tests increased the AUROCs to 0.79 for NASH and 0.96 for cirrhosis.^79^

The FIB-4 test combines age with three standard biochemical values (platelets, ALT and AST) to assess fibrosis. In NAFLD FIB-4 has demonstrated similar results to the AST/ALT ratio where, using a cut-off of 1.3, an AUROC of 0.86, sensitivity 85%, specificity 65% and NPV of 95% were demonstrated for the diagnosis of advanced fibrosis.^70^ In a US-based comparison of several non-invasive markers of fibrosis in 541 NAFLD patients, FIB-4 had the highest AUROC of 0.802, with PPV and NPV of 80% and 90% respectively for diagnosis of advanced fibrosis. In this study, AUROCs for the NAFLD fibrosis score, AAR, APRI, AST:platelet ratio and BARD score were 0.768, 0.742, 0.73, 0.72 and 0.70 respectively.^80^

Increased serum GGT level has also been shown to be associated with advanced fibrosis in NAFLD, with a study of 50 NAFLD patients demonstrating an AUROC of 0.74 for the prediction of advanced fibrosis. Using a cut-off serum GGT value of 96.5 U/L, GGT predicted advanced fibrosis with 83% sensitivity and 69% specificity.^81^

FibroMeter is a panel of serum markers which was originally developed for staging fibrosis in chronic HCV.^82^ However, FibroMeter NAFLD has since been developed which has shown good diagnostic accuracy in staging NASH-related fibrosis. This panel combines seven variables (age, weight, fasting glucose, AST, ALT, ferritin and platelet count), and in a study of 235 NAFLD patients demonstrated AUROCs of 0.943 for significant fibrosis, 0.937 for severe fibrosis and 0.904 for cirrhosis respectively. The sensitivity, specificity, PPV and NPV of FibroMeter for diagnosing significant fibrosis were 78.5%, 95.9%, 87.9 and 92.1%.^76^

The NAFLD fibrosis score (NFS) is a panel comprising six variables of age, hyperglycaemia, BMI, platelet count, albumin and AST/ALT ratio, which was constructed using a large panel of 733 biopsy-proven NAFLD patients across several centres worldwide. Two cut-off scores were generated to predict the likelihood of the presence or absence of advanced fibrosis respectively.^67^ In the original study, by applying the low cut-off score (−1.455), the NFS had an NPV of 93% and 88% in the estimation and validation groups respectively for excluding the presence of advanced fibrosis. By applying the high cut-off score (0.676), PPVs of 90% and 82% in the estimation and validation groups respectively were achieved for predicting the presence of advanced fibrosis. The AUROC was 0.84, and application of this model to the study population would have avoided liver biopsy in 75% of patients, with a correct prediction in 90%.^67^ In the recent study by McPherson *et al.*, the NFS demonstrated an AUROC of 0.81 with NPV 92% and PPV 72%, which was the highest PPV of the four tests examined.^70^ Cales *et al.* demonstrated an AUROC of 0.884 for significant fibrosis, 0.932 for severe fibrosis and 0.902 for cirrhosis.^76^ Studies in East Asian populations have also demonstrated good accuracy for excluding advanced fibrosis, with NPVs of 89% and 91% demonstrated in Japanese^72^ and Chinese^83^ NAFLD cohorts respectively. The NFS also demonstrated excellent accuracy at excluding fibrosis in morbidly obese subjects with NAFLD undergoing bariatric surgery, where NPVs of 98%, 87% and 88% for excluding advanced, significant and any fibrosis respectively were demonstrated.^84^ In a recent meta-analyses, NFS achieved pooled AUROC, sensitivity and specificity of 0.85 (0.80–0.93), 0.90 (0.82–0.99) and 0.97 (0.94–0.99) for the identification of NASH with advanced fibrosis.^85^ The NAFLD fibrosis score thus facilitates the identification of NAFLD patients with more advanced disease who require ongoing follow-up, and considerably reduces the requirement for liver biopsy in the minority of patients with an indeterminate score.^70^

Of these various algorithms FIB-4 and the NAFLD fibrosis score (NFS) have been validated most widely with demonstrably superior test characteristics.^70^, ^85^

*(ii) Fibrosis biomarkers:* The Original ELF (European Liver Fibrosis) test is a panel of automated immunoassays to detect three markers of matrix turnover in serum: hyaluronic acid (HA), tissue inhibitor of metalloproteinase 1 (TIMP1) and aminoterminal peptide of pro-collagen III (P3NP), used in combination with age.^86^ The simplified ELF panel excludes age but has a similar diagnostic performance. The addition of five simple markers – BMI, presence of diabetes/impaired fasting glucose, AST/ALT ratio, platelets and albumin – to the ELF test improved diagnostic accuracy further, with AUROCs of 0.98, 0.93 and 0.84 for the diagnosis of severe, moderate and no fibrosis respectively.^87^ The ELF panel may also represent a useful prognostic tool, with a one unit change in ELF score shown to be associated with a doubling of the odds of significant liver-related mortality or morbidity at 6 year follow-up.^88^

FibroTest is another validated marker for the quantitative assessment of fibrosis in NAFLD, ALD and chronic viral hepatitis.^89^ Combining five biochemical markers of haptoglobin, α2-macroglobulin, apolipoprotein A1, total bilirubin and GGT, corrected for age and gender, a mean standardised AUROC of 0.84 for advanced fibrosis in NAFLD patients was demonstrated using FibroTest in one meta-analyses. Importantly the diagnostic value was found to be similar for the diagnosis of both intermediate and extreme fibrosis stages, with no significant difference between the AUROC of the intermediate adjacent stages F2 vs. F1 to that of the extreme stages F3 vs. F4 or F1 vs. F0.^89^ FibroTest can also be combined with two other panels – SteatoTest and NASHTest – to form the FibroMax panel (BioPredictive, Paris, France), which provides a simultaneous and complete estimation of the liver injury in NAFLD.^89^ FibroTest/FibroMax have now been widely adopted as a non-invasive alternative to liver biopsy.^90^

Other potential fibrosis biomarkers include type VI collagen 7S domain and hyaluronic acid (HA), with the latter also representing a constituent of the ELF test. In a cohort of 112 NAFLD subjects, these two biomarkers were able to exclude advanced fibrosis with AUROCs of 0.82 and 0.80, and NPVs of 84 and 78% respectively. These biomarkers also demonstrated PPVs of 86% and 92% and AUROCs of 0.83 and 0.80 for discriminating NASH from simple fatty liver.^91^ In a separate cohort of 72 patients, the AUROC curves for type IV collagen 7S domain and HA were 0.767 and 0.754 respectively for the detection of advanced fibrosis in NASH. However, after multiple regression analysis only type IV collagen 7S domain was independently associated with advanced fibrosis in this study.^92^ Other studies of HA in NAFLD using varying cut-off levels have demonstrated AUROCs of between 0.89 and 0.97 for detecting advanced fibrosis.^93^–^95^ Kaneda *et al.* demonstrated HA to have an AUROC, NPV, sensitivity and specificity of 0.97, 100%, 100% and 89% respectively for detecting severe fibrosis, with a lower AUROC of 0.87 demonstrated for type IV collagen. In this study, the platelet count alone was an independent predictor of cirrhosis, with an AUROC of 0.98, and sensitivity, specificity, PPV and NPV of 100%, 95%, 76% and 100% respectively using a cut-off value of 16 × 10^4^/μL.^94^ Lesmana *et al.* also demonstrated the utility of levels of HA and type IV collagen to differentiate between mild (F1-2) and advanced fibrosis (F3-4),^96^ and in a separate study of 80 NAFLD patients the combination of HA with AST, AAR, age, gender and BMI demonstrated an AUROC of 0.763 for distinguishing simple steatosis and NASH.^97^

In a small study of serum extracellular matrix components in 30 NAFLD patients, serum laminin >282 ng/mL was shown to have an accuracy of 87%, sensitivity 82%, specificity 89%, PPV 82% and NPV 89% for identifying the presence of NASH with fibrosis. When combined with type IV collagen, both specificity and PPV increased to 100%, but with lower sensitivity and NPV of 64% and 83% respectively.^98^

*(iii) Radiological assessment:* Although many imaging modalities have been evaluated in NAFLD, their major focus has been quantification of hepatic fat, with few allowing a reliable distinction between simple steatosis and steatohepatitis or fibrosis.^52^, ^99^ Conventional imaging techniques include ultrasound (US), computed tomography (CT) and magnetic resonance imaging (MRI).

#### Fibroscan

Transient elastography (Fibroscan, Echosens, Paris, France) is a non-invasive method of assessing liver fibrosis which can be performed at the bedside or in the out-patient clinic. It employs ultrasound-based technology to measure liver stiffness (LSM), and has been validated for use in chronic hepatitis C, HIV/HCV coinfection and cholestatic liver diseases.^100^ Failure to obtain a reading occurs in only 5% of cases, but is more common in obese patients which has so far limited its use in the NAFLD cohort,^100^ although a recently introduced XL probe may reduce this problem.^101^ Although Fibroscan is less well validated in NAFLD, a stepwise increase in liver stiffness with increasing histological fibrosis was demonstrated in a study of 97 Japanese NAFLD patients, where AUROCs for the diagnosis of significant fibrosis, severe fibrosis and cirrhosis were 0.88, 0.91 and 0.99 respectively.^102^ A larger study including 246 NAFLD patients from two ethnic groups demonstrated AUROCs for the diagnosis of moderate fibrosis (≥F2), bridging fibrosis (≥F3) or cirrhosis (F4) of 0.84, 0.93 and 0.95 respectively.^103^ In this study, the best LSM cut-off scores for predicting F ≥ 2, F ≥ 3 and F4 were 7.0 kPa, 8.7 kPa and 10.3 kPa respectively. Cut-off values of 5.8 kPa and 9 kPa, and 7.9 kPa and 9.6 kPa had >90% sensitivity and specificity to rule out and rule in F2 and F3 fibrosis respectively. The cut-off of 10.3 kPa for F4 disease had 92% sensitivity and 88% specificity.^103^ In this study, if liver biopsy was reserved for those patients with LSM of ≥8.7 kPa, 32% would require the procedure, which would miss only 4.8% patients with F3 and 0.6% patients with cirrhosis. However, if biopsy was performed only in those with scores between 7.9 and 9.6 kPa, only 16% patients would require biopsy.^103^ In a 2010 meta-analyses of non-invasive assessment tools in NAFLD, transient elastography demonstrated pooled AUROC, sensitivity and specificity of 0.94 (0.90–0.99), 0.94 (0.88–0.99) and 0.95 (0.89–0.99).^85^ Although further studies will undoubtedly add to the current evidence base, Fibroscan has now been validated in NAFLD,^104^ and represents a useful tool for rapid, non-invasive assessment of liver fibrosis and determining need for biopsy.

An MR equivalent of transient elastography has recently demonstrated excellent diagnostic accuracy with sensitivity and specificity of 98% and 99% respectively for detecting all grades of fibrosis.^105^ MR elastography was also associated with a higher technical success rate than US elastography,^106^ and hepatic stiffness did not appear to be affected by steatosis using this technique^105^ which had been a previous concern.^107^ However, this technique remains experimental at the present time.

The combination of transient elastography with one or more of the serum marker panels described above represents a potential approach to the non-invasive measurement of fibrosis in NAFLD.^37^

#### Assessment of NASH inflammation and steatosis

*(i) Serum markers:* SteatoTest combines 10 readily available blood tests with age, gender and BMI, and in a study of >2000 patients with viral hepatitis, NAFLD or ALD, demonstrated an AUROC of 0.8 for the diagnosis of steatosis, which was superior to that of GGT, ALT or ultrasound.^90^, ^108^ NASHTest combines 13 biochemical and clinical variables to predict the presence or absence of NASH, achieving specificity, sensitivity, PPV and NPV of 94%, 33%, 66% and 81% respectively.^109^ Together with FibroTest, these three panels comprise the FibroMax panel described previously.^89^

Many other potential serum biomarkers have been identified which are typically markers of the key mechanisms believed to be involved in NASH pathogenesis, such as inflammation, oxidative stress, apoptosis and insulin resistance.^44^ Inflammation is associated with an increase in tumour necrosis factor-alpha (TNF-α) and decreased adiponectin expression (measured by ELISA) and this cytokine imbalance does appear to correlate with NASH,^110^–^112^ although the accuracy or clinical usefulness of these markers is yet to be determined.^113^ Shimada *et al.* observed the serum adiponectin level to be significantly lower in patients with early-stage NASH (3.6 μg/mL) than in those with simple steatosis (6.0 μg/mL), with an AUROC of 0.765, sensitivity 68% and sensitivity 79% for distinguishing early-stage NASH. In this study, the combination of serum adiponectin level with HOMA-IR and type IV collagen 7S demonstrated a sensitivity of 94% and specificity of 74% for diagnosing NASH.^114^ Hui *et al.* also reported significantly lower adiponectin levels and higher HOMA-IR in patients with NASH compared with subjects with simple steatosis, and demonstrated an AUROC of 0.79 for the combination of these markers for distinguishing between steatohepatitis and steatosis.^115^ However, the relationship between adiponectin levels and severity of hepatic fibrosis remains to be established.^116^, ^117^

The inflammatory marker C-reactive protein (CRP), measured using immunometric assay, has demonstrated mixed results in NASH. While some studies have shown a significant increase in high-sensitivity CRP levels in NASH patients compared with controls,^118^, ^119^ another study demonstrated no significant difference.^120^ CRP also lacks specificity for hepatic inflammation. Interleukin-6 (IL-6) is another marker of inflammation which has been shown to be elevated in NASH.^121^ In a study comparing 43 NASH patients, 40 subjects with steatosis and 48 controls, normal levels of IL-6 were highly specific in confirming the absence of NASH, with an AUROC of 0.817 for distinguishing NASH from simple steatosis. The same study also demonstrated significantly elevated levels of vascular endothelial growth factor (VEGF) concentrations in NASH, although the AUROC of 0.678 was lower than for IL-6.^122^ IL-6 levels were also independently associated with fibrosis in a study by Lemoine *et al.*, where the combination of HOMA-IR with the adiponectin/leptin ratio demonstrated an AUROC of 0.82 for distinguishing between NASH and simple steatosis.^123^

Indicators of oxidative stress, including lipid peroxidation products (measured using a spectrofluorometric method), vitamin E levels (measured using reverse phase high performance liquid chromatography), and copper-to-zinc superoxide dismutase and glutathione peroxidase (GSH-Px) activity (measured using commercial antioxidant assay kits), have been investigated as surrogate markers of NASH. However, most studies to date have been small with mixed results,^113^, ^124^, ^125^ and it is not yet clear whether or not oxidative stress in the liver is accurately reflected in the serum.^126^

Thioredoxin (TRX) is stress-inducible thiol-containing protein which may represent a clinically useful indicator of oxidative stress.^127^ In a small study of 57 patients, Sumida *et al.* demonstrated significantly elevated serum TRX levels in patients with NASH compared with those with simple steatosis and healthy controls, with an AUROC of 0.785 for distinguishing NASH from simple steatosis.^127^ Similar findings have been reported elsewhere, with a correlation between serum TRX and ferritin levels also observed.^128^

Apoptosis plays an important role in the liver injury observed in NAFLD,^129^ and cytokeratin-18 (CK-18) represents a useful marker of this process. In a US multi-centre validation study including 139 NAFLD patients and 150 controls, Feldstein *et al.* demonstrated that plasma CK-18 levels measured using ELISA were significantly higher in patients with biopsy-proven NASH than in those with a borderline diagnosis and normal controls, with an AUROC of 0.83 for NASH diagnosis. CK-18 was an independent predictor of both NASH and severity of disease.^130^ Other studies have corroborated these findings,^131^–^137^ suggesting that CK-18 represents a potentially useful biomarker for the diagnosis and differentiation of NASH from simple steatosis. CK-18 recently received independent validation for diagnosing NASH in a 2010 meta-analyses, where pooled AUROC, sensitivity and specificity for NASH were 0.82 (0.78–0.88), 0.78 (0.64–0.92), and 0.87 (0.77–0.98) respectively.^85^ An AUROC of 0.88 for diagnosis of NASH was also demonstrated in a morbidly obese population where, in addition, CK-18 levels were observed to fall significantly following bariatric surgery.^138^ Younossi *et al.* evaluated the diagnostic utility of several ELISA-based assays in patients with biopsy-proven NASH. This study found that the levels of cleaved CK-18 (M30 antigen), and intact CK-18 (M65) predicted histological NASH with 70% sensitivity, 84% specificity, AUROC 0.711 and 64% sensitivity, 89% specificity and AUROC 0.814 respectively. Histological NASH was found to be predicted by a combination of four ELISA-based tests – cleaved CK-18, a product of the subtraction of cleaved CK-18 level from intact CK-18 level, serum adiponectin and serum resistin – with a sensitivity 96%, specificity of 70% and AUROC of 0.91.^139^ CK18 fragment has also been combined with ALT levels and the presence of the metabolic syndrome in the ‘Nice model’, a composite model where AUROCs of 0.83–0-88 were demonstrated for the diagnosis of NASH, as defined by a NAS score ≥5, in a morbidly obese population.^140^

Plasma homocysteine (Hcy) levels were shown to distinguish NASH from simple steatosis with good accuracy in a Turkish study of 71 NAFLD patients. Using a threshold of 11.935 ng/mL, sensitivity and specificity were 91.7% and 95.7% with an AUROC of 0.948 for predicting NASH.^141^

Serum prolidase enzyme activity (SPEA) catalyses the final step of collagen breakdown by liberating free proline for collagen recycling, and is reported to be of hepatic origin.^142^ In a Turkish study of 54 NAFLD patients, SPEA was significantly greater in patients with NASH than those with simple steatosis or controls, and positively correlated with the grade of liver fatty infiltration, lobular inflammation, stage of fibrosis and NAFLD activity score.^142^ In this study, the SPEA was shown to be superior to AST, ALT or the AAR for distinguishing NASH from simple steatosis, with an AUROC of 0.85, and also the most useful of these tests for predicting lobular inflammation, NAFLD activity score and fibrosis.^142^ Other novel biomarkers which may be of potential utility in the diagnosis of NASH include plasma pentraxin 3 levels^143^ and tissue polypeptide specific antigen.^144^ Further study of these markers is warranted.

It should be highlighted that although some of the non-invasive serum markers described earlier are already widely used in the assessment and staging of NASH, further validation of their use in this setting will increase confidence in their utility (Table 2).

**Table 2:** Non-invasive techniques for diagnosis of (a) NASH and (b) fibrosis in patients with NAFLD in descending order of diagnostic accuracy

a)		

Diagnostic technique	Size of cohort used in studies/Comments	Popularity
Contrast-enhanced ultrasound	Study including 64 patients with normal liver, NAFLD or NASH, demonstrated AUROC of 100% for diagnosis of NASH^145^	Use currently limited to clinical trials
NASHTest	Study of 257 patients: 160 in training group and 97 in validation group. Specificity, sensitivity, PPV and NPV of 94%, 33%, 66% and 81% for diagnosis of NASH. Can be combined with SteatoTest and FibroTest in Fibromax^109^	Use mainly confined to specialist liver centres
CK-18	Multi-centre validation study including 139 NAFLD patients demonstrated AUROC of 0.83 for NASH diagnosis.^130^ Recently received independent validation for diagnosing NASH in meta-analyses demonstrating pooled AUROC, sensitivity and specificity for NASH of 0.82, 0.78 and 0.87^85^	Use currently limited to clinical trials but holds promise for more widespread application

b)		

Diagnostic technique	Size of cohort used in studies/Comments	Popularity
ELF test + simple markers	Validation study included 196 NAFLD patients. AUROCs of 0.84, 0.93 and 0.98 for detecting no fibrosis, moderate fibrosis and severe fibrosis respectively^87^	Use mainly confined to specialist liver centres
FibroMeter	Study of 235 NAFLD patients demonstrated AUROCs of 0.943 for significant fibrosis, 0.937 for severe fibrosis and 0.904 for cirrhosis respectively. Sensitivity, specificity, PPV, NPV for diagnosis of significant fibrosis were 79%, 96%, 88% and 92%^76^	Use mainly confined to specialist liver centres
Fibroscan	Largest study included 246 NAFLD patients, with AUROCs of 0.84, 0.93 and 0.95 for diagnosis of ≥F2, ≥F3 and ≥F4 fibrosis respectively.^103^ Meta-analyses of non-invasive assessment tools in NAFLD demonstrated pooled AUROC, sensitivity and specificity of 0.94, 0.94 and 0.95^85^	Use mainly confined to specialist liver centres. Equipment expensive
NAFLD Fibrosis Score (NFS)	Validation study included 733 patients with biopsy-proven NAFLD: 480 in estimation group and 253 in validation group. PPVs of 90% and 82% for predicting advanced fibrosis and NPVs of 93% and 88% for excluding advanced fibrosis in estimation and validation groups respectively, with AUROC 0.84^67^	Widely used in secondary care clinical practice. Accessibility increased by availability of on-line calculator. Use advocated in recent review of non-invasive scoring systems for exclusion of advanced fibrosis^70^
	For diagnosis of advanced fibrosis, AUROC of 0.768 demonstrated in study of 541 patients,^80^ and AUROC of 0.81 in study of 145 NAFLD patients.^70^ In study of 235 patients, AUROCs of 0.884 for significant fibrosis, 0.932 for severe fibrosis, and 0.902 for cirrhosis^76^	
	Similar efficacy demonstrated in East Asian^72^, ^83^ and morbidly obese cohorts^84^	
FibroTest	Meta-analyses including 267 NAFLD patients demonstrated mean AUROC of 0.84 for detecting advanced fibrosis. Can be combined with SteatoTest and NASHTest in Fibromax^89^	Use mainly confined to specialist liver centres
FIB-4	Study of 541 NAFLD patients demonstrated AUROC of 0.802, with PPV 80% and NPV 90% for diagnosis of advanced fibrosis.^80^ Study of 145 NAFLD patients demonstrated AUROC of 0.86, sensitivity 85%, specificity 65% and NPV of 95%^70^	Use advocated in recent review of non-invasive scoring systems for exclusion of advanced fibrosis^70^
APRI (AST-to-platelet ratio index)	Study of 235 NAFLD patients demonstrated AUROCs of 0.866 for significant fibrosis, 0.861 for severe fibrosis and 0.842 for cirrhosis.^76^ AUROCs of 0.73 for advanced fibrosis in study of 541 NAFLD patients,^80^ and 0.67 in study of 145 patients^70^	Developed for use in HCV. Not specific for NAFLD
AST/ALT ratio (AAR)	Using cut-off of 0.8, study of 145 NAFLD patients demonstrated AUROC of 0.83, sensitivity 74%, specificity 78% and NPV 93% for diagnosis of advanced fibrosis.^70^ AUROC 0.742 for advanced fibrosis in study of 541 NAFLD patients^80^	Easy to calculate. Also a component of some other fibrosis scoring systems eg. NFS, BARD. Use advocated in recent review of non-invasive scoring systems for exclusion of advanced fibrosis^70^
BARD score	Original study included 827 NAFLD patients with NPV 96% for excluding advanced fibrosis.^68^ AUROC 0.70 for advanced fibrosis in study of 541 NAFLD patients.^80^ Study of 145 patients showed AUROC of 0.77, with sensitivity 89%, specificity 44%, NPV 95% and PPV 25%^70^	Simple to use in primary or secondary care. Use advocated in recent review of non-invasive scoring systems for exclusion of advanced fibrosis^70^

Techniques to non-invasively distinguish NASH from simple steatosis remain largely experimental, although show much promise for the future. Several methods are available for the non-invasive diagnosis/exclusion of fibrosis in patients with NAFLD. The BARD score is the simplest scoring system to calculate in the clinic, but the NAFLD fibrosis score can also be easily calculated by entering the relevant details into a freely available online calculator^70^ (http://nafldscore.com). Currently FibroTest/FibroMax, FibroMeter and the ELF test are each only available from a single laboratory at significant cost, which has limited their use to mainly specialist liver centres.

*(ii) Radiological assessment:* Contrast-enhanced ultrasound using Levovist is the first imaging technique to demonstrate efficacy in distinguishing between simple steatosis and NASH. In a study of 64 patients with either normal liver, NAFLD or NASH, this modality was able to diagnose NASH with an AUROC of 100%.^145^ The accumulation of Levovist microbubbles in the liver parenchyma was shown to be decreased in NASH but not in NAFLD or chronic viral hepatitis, with the decrease seen in NASH correlating with fibrosis rather than steatosis. Severe decrease was seen in both NASH and ASH livers, especially in the presence of bridging fibrosis (F3); however, the same stage of fibrosis in chronic viral hepatitis showed only a mild decrease or normal uptake of Levovist. These differences may be explained both by changes in Kupffer cell function and differences between pericellular and periportal fibrosis in provoking disturbance of Levovist microbubble accumulation.^145^ Although this remains an experimental technique at present, evolving imaging modalities may thus soon permit the non-invasive differentiation between steatosis and NASH.

The diagnostic yield using CT is similar to that of US in NAFLD. Unenhanced CT shows low attenuation of the steatotic liver in contrast to the spleen, and the severity of steatosis has been shown to correlate with the liver:spleen (L/S) attenuation ratio.^146^ A CT L/S cut-off of value of 0.8 yielded 100% specificity and 82% sensitivity for diagnosing macrovesicular steatosis of 30% or greater,^147^ although a CT L/S cut off of 1.0 for defining steatosis has been used in some studies.^148^ Accuracy of unenhanced CT is greatly reduced with lesser degrees of steatosis.^147^ Other pathologies, such as hepatic siderosis, may also alter attenuation values leading to misdiagnosis,^149^, ^150^ and the radiation exposure associated with CT limits its use in younger patients and in longitudinal studies.^149^

Fibroscan has also recently demonstrated utility in detecting and quantifying steatosis, using a novel attenuation parameter termed ‘Controlled Attenuation Parameter’ (CAP), which was devised to specifically target the liver using a process based on Vibration Control Transient Elastography (VCTE, Echosens, Paris, France). A study of 115 patients using liver biopsy as reference demonstrated that CAP was able to accurately detect >10% (S1), >33% (S2) and >67% (S3) steatosis with AUROCs of 0.91, 0.95 and 0.89 respectively. CAP evaluated by the Fibroscan is not affected by fibrosis, and advantages over other imaging techniques include its ability to quantify and detect steatosis from only 10% of liver infiltration, and being non-ionising, relatively cheap and non-operator dependent.^151^

**Figure 2:**
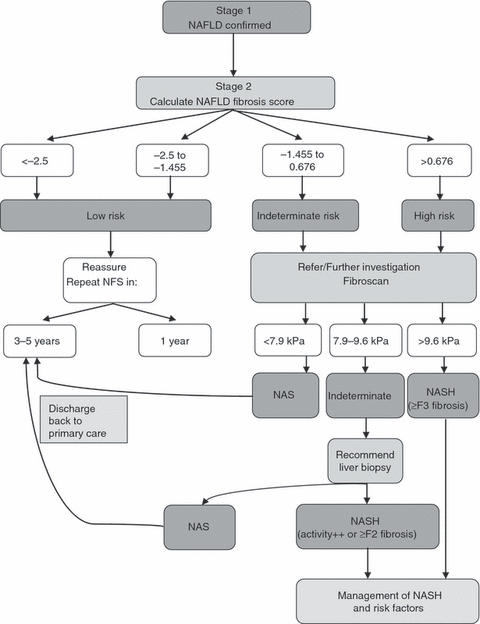
Proposed algorithm for the work-up of a patient with NAFLD. Patients with a NAFLD fibrosis score below the lower cut-off level have a low risk of significant fibrosis and subsequent disease progression and can be safely managed in primary care. Referral to specialist care is indicated if disease progression is suspected on clinical or biochemical grounds. A score in the indeterminate range or above merits further investigation by use of modalities such as specialist scans or blood tests. Liver biopsy should be considered for those patients in whom non-invasive tests are inconclusive. The use of Fibroscan in this algorithm may later be replaced by serum marker panels. NFS, NAFLD fibrosis score; NAS, non-alcoholic steatosis.

There are at present no evidence-based guidelines for the work-up/staging of a patient with confirmed NAFLD. Although alternative approaches may be equally effective, a proposed algorithm for the work-up of patients with NAFLD is detailed in Figure 2. This combination of inexpensive non-invasive algorithms prior to more detailed examinations should hopefully permit a cost-effective and efficient way of investigating what is likely to be a large number of potential patients.

## Conclusions

Non-alcoholic fatty liver disease now represents the most common cause of liver disease in the Western world, and rising levels of obesity, diabetes and the metabolic syndrome render it an increasingly important cause of morbidity and mortality. While simple steatosis carries a relatively benign prognosis, a significant proportion of patients will progress to NASH and later cirrhosis with risk of hepatocellular carcinoma. Although liver biopsy remains the gold standard for disease assessment, the development of risk scores, biomarker panels and ultrasound modalities has resulted in much improved identification of at risk patients without recourse to use of liver biopsy on a routine basis.
